# Supplementary material for: Stochastic gradient descent estimation of generalized matrix factorization models with application to single-cell RNA sequencing data
Source: Biostatistics. 2026 May 4;27(1):kxag010. doi: 10.1093/biostatistics/kxag010 (PMC13143404; doi:10.1093/biostatistics/kxag010)
Supplement: kxag010_Supplementary_Data [file kxag010_supplementary_data.pdf]

Biostatistics (2026), **0**, 0, *pp.* 1–**??**
doi:10.1093/biostatistics/output

Supplementary Materials for "Stochastic gradient descent estimation of generalized matrix factorization models with application to single-cell RNA sequencing data"

CRISTIAN CASTIGLIONE[1], ALEXANDRE SEGERS[2], LIEVEN CLEMENT[2], DAVIDE RISSO[3,*]

[1] *Institute for Data Science and Analytics, Bocconi University, Via Röntgen 1, 20136, Milan, Italy*

[2] *Department of Applied Mathematics, Computer Science and Statistics, Ghent University, Krijgslaan 281-S9, 9000, Ghent, Belgium*

[3] *Department of Statistical Sciences, University of Padova, Via Cesare Battisti 241, 35121, Padova, Italy*

Corresponding author: Department of Statistical Sciences, University of Padova, Via Cesare Battisti 241, 35121, Padova, Italy. Email: davide.risso@unipd.it

1. Parameter identifiability

Here, we discuss the set of identifiability constraints introduced in Section 2.1, proving the model identification under constraints (A) and (B), and providing a practical and efficient post-processing approach to enforce the identification of the parameter estimates.

Proposition 1 Under assumption (A) and any of the equivalent conditions (B1), (B2), or (B3),

© The Author 2026. Published by Oxford University Press. All rights reserved. For permissions, please e-mail: journals.permissions@oup.com

the GMF model in (2.1)–(2.3) is computationally identifiable.

To streamline the proof of Proposition 1, we first state and demonstrate the following Lemmas.

LEMMA 1.1 Let $(\mathbf{B}_1, \mathbf{\Gamma}_1, \mathbf{U}_1, \mathbf{V}_1)$ and $(\mathbf{B}_2, \mathbf{\Gamma}_2, \mathbf{U}_2, \mathbf{V}_2)$ be two configurations of the parameters, both satisfying (A) and yielding the same linear predictor. Then $\mathbf{B}_1 = \mathbf{B}_2$, $\mathbf{\Gamma}_1 = \mathbf{\Gamma}_2$, and $\mathbf{U}_1\mathbf{V}_1^\top = \mathbf{U}_2\mathbf{V}_2^\top$.

Proof. By hypothesis, $(\mathbf{B}_1, \mathbf{\Gamma}_1, \mathbf{U}_1, \mathbf{V}_1)$ and $(\mathbf{B}_2, \mathbf{\Gamma}_2, \mathbf{U}_2, \mathbf{V}_2)$ yield the same linear predictor, that is

$$\boldsymbol{\eta}_1 = \mathbf{X}\mathbf{B}_1^\top + \mathbf{\Gamma}_1\mathbf{Z}^\top + \mathbf{U}_1\mathbf{V}_1^\top \;=\; \mathbf{X}\mathbf{B}_2^\top + \mathbf{\Gamma}_2\mathbf{Z}^\top + \mathbf{U}_2\mathbf{V}_2^\top = \boldsymbol{\eta}_2. \tag{1.1}$$

Left-multiplying both sides of (1.1) by $\mathbf{X}^\top$, and using of the constraints $\mathbf{X}^\top\mathbf{\Gamma}_1 = \mathbf{X}^\top\mathbf{\Gamma}_2 = \mathbf{0}$ and $\mathbf{X}^\top\mathbf{U}_1 = \mathbf{X}^\top\mathbf{U}_2 = \mathbf{0}$, we obtain $(\mathbf{X}^\top\mathbf{X})\mathbf{B}_1 = (\mathbf{X}^\top\mathbf{X})\mathbf{B}_2$. Thanks to the non-singularity of $\mathbf{X}^\top\mathbf{X}$, we conclude that $\mathbf{B}_1 = \mathbf{B}_2$.

Subtracting the common term $\mathbf{X}\mathbf{B}_1^\top$ (equal to $\mathbf{X}\mathbf{B}_2^\top$) from both sides of (1.1), we obtain $\mathbf{\Gamma}_1\mathbf{Z}^\top + \mathbf{U}_1\mathbf{V}_1^\top = \mathbf{\Gamma}_2\mathbf{Z}^\top + \mathbf{U}_2\mathbf{V}_2^\top$. Right-multiplying by $\mathbf{Z}$, and using the constraints $\mathbf{Z}^\top\mathbf{V}_1 = \mathbf{Z}^\top\mathbf{V}_2 = \mathbf{0}$, we get $\mathbf{\Gamma}_1(\mathbf{Z}^\top\mathbf{Z}) = \mathbf{\Gamma}_2(\mathbf{Z}^\top\mathbf{Z})$. This, along with the non-singularity of $\mathbf{Z}^\top\mathbf{Z}$, implies $\mathbf{\Gamma}_1 = \mathbf{\Gamma}_2$.

Moreover, given the identities $\mathbf{B}_1 = \mathbf{B}_2$ and $\mathbf{\Gamma}_1 = \mathbf{\Gamma}_2$, equation (1.1) entails $\mathbf{U}_1\mathbf{V}_1^\top = \mathbf{U}_2\mathbf{V}_2^\top$. This concludes the proof. □

LEMMA 1.2 Let $(\mathbf{U}_1, \mathbf{V}_1)$ and $(\mathbf{U}_2, \mathbf{V}_2)$ be two configurations of the score and loading parameters, both satisfying either (B1) or (B2), and $\mathbf{U}_1\mathbf{V}_1^\top = \mathbf{U}_2\mathbf{V}_2^\top$. Then $\mathbf{U}_1 = \mathbf{U}_2$ and $\mathbf{V}_1 = \mathbf{V}_2$.

Proof. First, we consider assumption (B1). Right-multiplying $\mathbf{U}_1\mathbf{V}_1^\top = \mathbf{U}_2\mathbf{V}_2^\top$ on both sides by $\mathbf{V}_1$, and exploiting $\mathbf{V}_1^\top\mathbf{V}_1 = \mathbf{I}_d$, we obtain $\mathbf{U}_1 = \mathbf{U}_2(\mathbf{V}_2^\top\mathbf{V}_1)$. Let $\mathbf{A} = \mathbf{V}_2^\top\mathbf{V}_1$, then, substituting $\mathbf{U}_1 = \mathbf{U}_2\mathbf{A}$ into $\mathbf{U}_1\mathbf{V}_1^\top = \mathbf{U}_2\mathbf{V}_2^\top$, left-multiplying the result by $\mathbf{U}_2^\top$, and using the non-singularity

of $\mathbf{U}_2^\top \mathbf{U}_2$, we obtain $\mathbf{V}_2 = \mathbf{V}_1 \mathbf{A}^\top$.

Sequentially applying $\mathbf{V}_2^\top \mathbf{V}_2 = \mathbf{I}_d$, $\mathbf{V}_2 = \mathbf{V}_1 \mathbf{A}^\top$, and $\mathbf{V}_1^\top \mathbf{V}_1 = \mathbf{I}_d$, we have

$$\mathbf{I}_d = \mathbf{V}_2^\top \mathbf{V}_2 = \mathbf{A} \mathbf{V}_1^\top \mathbf{V}_1 \mathbf{A}^\top = \mathbf{A} \mathbf{I}_d \mathbf{A}^\top = \mathbf{A} \mathbf{A}^\top,$$

which implies that $\mathbf{A}$ is an orthogonal matrix.

Similarly, we sequentially use $\mathbf{U}_1^\top \mathbf{U}_1 = \mathbf{\Sigma}$, $\mathbf{U}_1 = \mathbf{U}_2 \mathbf{A}$, and $\mathbf{U}_2^\top \mathbf{U}_2 = \mathbf{\Sigma}$ to get

$$\mathbf{\Sigma} = \mathbf{U}_1^\top \mathbf{U}_1 = \mathbf{A}^\top \mathbf{U}_2^\top \mathbf{U}_2 \mathbf{A} = \mathbf{A}^\top \mathbf{\Sigma} \mathbf{A}.$$

Thus, $\mathbf{A}^\top \mathbf{\Sigma} \mathbf{A}$ is the eigenvalue decomposition of $\mathbf{\Sigma}$, where $\mathbf{A}$, due to the orthogonality, must be a diagonal sign matrix whose diagonal entries can only take values -1 or $+1$. Finally, since $\mathbf{V}_2 = \mathbf{V}_1 \mathbf{A}^\top$ and the first non-zero entry of each column of $\mathbf{V}_1$ and $\mathbf{V}_2$ must be positive because of (B1), we have $\mathbf{A} = \mathbf{I}_d$. As a consequence, $\mathbf{U}_1 = \mathbf{U}_2$ and $\mathbf{V}_1 = \mathbf{V}_2$. This concludes the proof for (B1).

The proof under (B2) trivially follows from the same argument. □

LEMMA 1.3 Let $(\mathbf{U}_1, \mathbf{V}_1)$ and $(\mathbf{U}_2, \mathbf{V}_2)$ be two configurations of the score and loading parameters, both satisfying (B3) and $\mathbf{U}_1 \mathbf{V}_1^\top = \mathbf{U}_2 \mathbf{V}_2^\top$. Then $\mathbf{U}_1 = \mathbf{U}_2$ and $\mathbf{V}_1 = \mathbf{V}_2$.

Proof. Following the same reasoning used in the proof of Lemma 1.2, and defining $\mathbf{H}_n = \frac{1}{n}(\mathbf{I}_n - \frac{1}{n}\mathbf{1}_n \mathbf{1}_n^\top)$, we can left-multiply $\mathbf{U}_1 \mathbf{V}_1^\top = \mathbf{U}_2 \mathbf{V}_2^\top$ on both sides by $\mathbf{U}_1^\top \mathbf{H}_n$ and use $\mathbf{U}_1^\top \mathbf{H}_n \mathbf{U}_1 = \mathbf{I}_d$ to obtain the identity $\mathbf{V}_1 = \mathbf{V}_2 \mathbf{A}^\top$, where $\mathbf{A} = \mathbf{U}_1^\top \mathbf{H}_n \mathbf{U}_2$. Substituting $\mathbf{V}_1 = \mathbf{V}_2 \mathbf{A}^\top$ into $\mathbf{U}_1 \mathbf{V}_1^\top = \mathbf{U}_2 \mathbf{V}_2^\top$, right-multiplying by $\mathbf{V}_2$, and exploiting the non-singularity of $\mathbf{V}_2^\top \mathbf{V}_2$, we obtain $\mathbf{U}_2 = \mathbf{U}_1 \mathbf{A}$. Hence, sequentially applying $\mathbf{U}_2^\top \mathbf{H}_n \mathbf{U}_2 = \mathbf{I}_d$, $\mathbf{U}_2 = \mathbf{U}_1 \mathbf{A}$, and $\mathbf{U}_1^\top \mathbf{H}_n \mathbf{U}_1 = \mathbf{I}_d$, we have

$$\mathbf{I}_d = \mathbf{U}_2^\top \mathbf{H}_n \mathbf{U}_2 = \mathbf{A}^\top \mathbf{U}_1^\top \mathbf{H}_n \mathbf{U}_1 \mathbf{A} = \mathbf{A}^\top \mathbf{I}_d \mathbf{A} = \mathbf{A}^\top \mathbf{A},$$

which entails that $\mathbf{A}$ is orthogonal.

Now, we know that $\mathbf{V}_1 = \mathbf{V}_2\mathbf{A}^\top$, where $\mathbf{V}_1$ and $\mathbf{V}_2$ are $m \times d$ matrices with zero upper-triangular part, and positive diagonal. Then, defining $\mathbf{R}_1$ and $\mathbf{R}_2$ as the $d \times d$ submatrices containing the first d rows of $\mathbf{V}_1$ and $\mathbf{V}_2$, respectively, we have $\mathbf{R}_1 = \mathbf{R}_2\mathbf{A}^\top$. By construction, $\mathbf{R}_1$ and $\mathbf{R}_2$ are non-singular squared lower-triangular matrices; thus we can write $\mathbf{R}_2^{-1}\mathbf{R}_1 = \mathbf{A}^\top$. As $\mathbf{R}_2$ and $\mathbf{R}_1$ are lower-triangular with positive diagonal entries, $\mathbf{R}_2^{-1}\mathbf{R}_1$ also inherits the same properties, as well as $\mathbf{A}^\top$, which is also orthogonal. Recall that orthogonal triangular matrices are, in fact, diagonal sign matrices, then $\mathbf{A}$ is diagonal with -1 or $+1$ diagonal entries. But $\mathbf{A}^\top = \mathbf{R}_2^{-1}\mathbf{R}_1$ must also have positive diagonal entries, hence $\mathbf{A} = \mathbf{I}_d$. And, finally, we can establish that $\mathbf{U}_1 = \mathbf{U}_2$ and $\mathbf{V}_1 = \mathbf{V}_2$. This concludes the proof. □

Now, we are ready to prove Proposition 1.

Proof of Proposition 1. The likelihood function of model (2.1)–(2.3)) depends on the parameters $(\mathbf{B}, \mathbf{\Gamma}, \mathbf{U}, \mathbf{V})$ solely through the linear predictor (2.3). Then, if we can prove that, under (A) and (B), equal linear predictors imply equal parameter values, this automatically ensures identifiability.

Suppose that $(\mathbf{B}_1, \mathbf{\Gamma}_1, \mathbf{U}_1, \mathbf{V}_1)$ and $(\mathbf{B}_2, \mathbf{\Gamma}_2, \mathbf{U}_2, \mathbf{V}_2)$ are two parameter configurations, both satisfying assumption (A) and yielding the same linear predictor. Then, thanks to assumption (A) and Lemma 1.1, we have $\mathbf{B}_1 = \mathbf{B}_2$, $\mathbf{\Gamma}_1 = \mathbf{\Gamma}_2$, and $\mathbf{U}_1\mathbf{V}_1^\top = \mathbf{U}_2\mathbf{V}_2^\top$. This means that assumption (A) alone is enough to ensure the identifiability of $\mathbf{B}$ and $\mathbf{\Gamma}$. Now, we are left to prove the identifiability of $\mathbf{U}$ and $\mathbf{V}$ under one of the equivalent additional restrictions (B1), (B2), and (B3).

Assuming that $(\mathbf{U}_1, \mathbf{V}_1)$ and $(\mathbf{U}_2, \mathbf{V}_2)$ satisfy both (A) and (B1), from Lemma 1.2 we have $\mathbf{U}_1 = \mathbf{U}_2$ and $\mathbf{V}_1 = \mathbf{V}_2$. The same holds under (A) and (B2). This ensures the identifiability of $(\mathbf{B}, \mathbf{\Gamma}, \mathbf{U}, \mathbf{V})$ under assumptions (A) and (B1), or (A) and (B2).

Finally, assuming that $(\mathbf{U}_1, \mathbf{V}_1)$ and $(\mathbf{U}_2, \mathbf{V}_2)$ satisfy both (A) and (B3), from Lemma 1.3 we have $\mathbf{U}_1 = \mathbf{U}_2$ and $\mathbf{V}_1 = \mathbf{V}_2$. This ensures the identifiability of $(\mathbf{B}, \mathbf{\Gamma}, \mathbf{U}, \mathbf{V})$ under assumptions

(A) and (B3). This concludes the proof. □

To obtain identifiable estimates that satisfy (A) and any of (B1), (B2), or (B3), we need a stable and efficient post-processing method to project unrestricted solutions onto the constrained space induced by the identifiability restrictions. To this end, we can apply standard projection methods. Consider, for instance, the constraint $\mathbf{X}^\top \mathbf{\Gamma} = \mathbf{0}$, and define $\mathbf{P}_{\text{X}} = \mathbf{X}(\mathbf{X}^\top \mathbf{X})^{-1}\mathbf{X}^\top$ as the projection matrix onto the column space of $\mathbf{X}$. As it is evident, $\mathbf{\Gamma} = \mathbf{P}_{\text{X}}\mathbf{\Gamma} + (\mathbf{I}_n - \mathbf{P}_{\text{X}})\mathbf{\Gamma}$, hence

$$\begin{aligned}
\mathbf{X}\mathbf{B}^\top + \mathbf{\Gamma}\mathbf{Z}^\top &= \mathbf{X}\mathbf{B}^\top + \mathbf{P}_{\text{X}}\mathbf{\Gamma}\mathbf{Z}^\top + (\mathbf{I}_n - \mathbf{P}_{\text{X}})\mathbf{\Gamma}\mathbf{Z}^\top \\
&= \mathbf{X}\mathbf{B}^\top + \mathbf{X}\big[(\mathbf{X}^\top\mathbf{X})^{-1}\mathbf{X}^\top\mathbf{\Gamma}\mathbf{Z}^\top\big] + \big[\mathbf{I}_n - \mathbf{X}(\mathbf{X}^\top\mathbf{X})^{-1}\mathbf{X}^\top\big]\mathbf{\Gamma}\mathbf{Z}^\top \\
&= \mathbf{X}\big[\mathbf{B} + \mathbf{Z}\mathbf{\Gamma}^\top\mathbf{X}(\mathbf{X}^\top\mathbf{X})^{-1}\big]^\top + \big[\mathbf{\Gamma} - \mathbf{X}(\mathbf{X}^\top\mathbf{X})^{-1}\mathbf{X}^\top\mathbf{\Gamma}\big]\mathbf{Z}^\top.
\end{aligned}$$

Therefore, we can reparametrize both $\mathbf{B}$ and $\mathbf{\Gamma}$ as follows

$$\mathbf{B}^* = \mathbf{B} + \mathbf{Z}\big[(\mathbf{X}^\top\mathbf{X})^{-1}\mathbf{X}^\top\mathbf{\Gamma}\big]^\top, \quad \mathbf{\Gamma}^* = \mathbf{\Gamma} - \mathbf{X}\big[(\mathbf{X}^\top\mathbf{X})^{-1}\mathbf{X}^\top\mathbf{\Gamma}\big].$$

In this way, by construction, $\mathbf{\Gamma}^*$ lies on the orthogonal complement of the column space of $\mathbf{X}$, thus $\mathbf{X}^\top\mathbf{\Gamma}^* = \mathbf{0}$. Of course, we also need to transform $\mathbf{B}$ to ensure that the identity $\mathbf{X}\mathbf{B} + \mathbf{\Gamma}\mathbf{Z}^\top = \mathbf{X}\mathbf{B}^{*\top} + \mathbf{\Gamma}^*\mathbf{Z}^\top$ holds true.

The same approach can be used to project $\mathbf{U}$ and $\mathbf{V}$ onto the orthogonal complement of the column space of $\mathbf{X}$ and $\mathbf{Z}$, respectively. Then, an effective algorithm to obtain orthogonality with respect to the covariate column space is to sequentially project $\mathbf{\Gamma}$, $\mathbf{U}$, and $\mathbf{V}$ onto the appropriate orthogonal complement, as shown in the first three rows of Algorithm 1.

Finally, to enforce one of (B1) or (B2), we can use standard reparametrizations based on the singular value decomposition of $\mathbf{U}\mathbf{V}^\top$ (see, e.g., Risso *and others*, 2018; Liu and Zhong, 2024). While (B3) requires first to rotate $\mathbf{U}$ and $\mathbf{V}$ using a whitening matrix, and then to triangularize $\mathbf{V}$ using the QR decomposition (see, e.g., Kidziński *and others*, 2022). A detailed pseudo-code description of all the projection steps and the relative computational costs is outlined in Algorithm 1.

Algorithm 1 Pseudo-code description of the post-processing algorithm used to project an unrestricted estimate of the GMF model parameters onto the constrained space induced by the identifiability conditions (A), (B1), (B2), and (B3). On the right, we report the computational complexity of each step. The compact notation svd() and qr() stand for the singular value and QR decomposition, respectively.

$\mathbf{D}_\Gamma \leftarrow (\mathbf{X}^\top\mathbf{X})^{-1}\mathbf{X}^\top\mathbf{\Gamma};\quad \mathbf{\Gamma} \leftarrow \mathbf{\Gamma} - \mathbf{X}\mathbf{D}_\Gamma;\quad \mathbf{B} \leftarrow \mathbf{B} + \mathbf{Z}\mathbf{D}_\Gamma^\top;$ — $O(p^3 + np^2 + npq)$

$\mathbf{D}_U \leftarrow (\mathbf{X}^\top\mathbf{X})^{-1}\mathbf{X}^\top\mathbf{U};\quad \mathbf{U} \leftarrow \mathbf{U} - \mathbf{X}\mathbf{D}_U;\quad \mathbf{B} \leftarrow \mathbf{B} + \mathbf{V}\mathbf{D}_U^\top;$ — $O(p^3 + np^2 + npd)$

$\mathbf{D}_V \leftarrow (\mathbf{Z}^\top\mathbf{Z})^{-1}\mathbf{Z}^\top\mathbf{V};\quad \mathbf{V} \leftarrow \mathbf{V} - \mathbf{Z}\mathbf{D}_V;\quad \mathbf{\Gamma} \leftarrow \mathbf{\Gamma} + \mathbf{U}\mathbf{D}_V^\top;$ — $O(q^3 + mq^2 + mqd)$

if (B1) **then**

  $\tilde{\mathbf{U}}, \tilde{\mathbf{\Sigma}}, \tilde{\mathbf{V}} \leftarrow \mathrm{svd}(\mathbf{U}\mathbf{V}^\top);\quad \mathbf{U} \leftarrow \tilde{\mathbf{U}}\tilde{\mathbf{\Sigma}};\quad \mathbf{V} \leftarrow \tilde{\mathbf{V}};$ — $O(nmd + nd + md)$

else if (B2) **then**

  $\tilde{\mathbf{U}}, \tilde{\mathbf{\Sigma}}, \tilde{\mathbf{V}} \leftarrow \mathrm{svd}(\mathbf{U}\mathbf{V}^\top);\quad \mathbf{U} \leftarrow \tilde{\mathbf{U}};\quad \mathbf{V} \leftarrow \tilde{\mathbf{V}}\tilde{\mathbf{\Sigma}};$ — $O(nmd + nd + md)$

else if (B3) **then**

  $\mathbf{S} \leftarrow \frac{1}{n}(\mathbf{U}^\top\mathbf{U} - \frac{1}{n}\mathbf{U}^\top\mathbf{1}_n\mathbf{1}_n^\top\mathbf{U});$ — $O(nd^2 + nd + d^2)$

  $\mathbf{U} \leftarrow \mathbf{U}\mathbf{S}^{-1/2};\quad \mathbf{V} \leftarrow \mathbf{V}\mathbf{S}^{1/2};$ — $O(nd^2 + md^2 + d^3)$

  $\mathbf{Q}, \mathbf{R} \leftarrow \mathrm{qr}(\mathbf{V}^\top);\quad \mathbf{U} \leftarrow \mathbf{U}\mathbf{Q};\quad \mathbf{V} \leftarrow \mathbf{R}^\top;$ — $O(md^2 + nd^2)$

end if

$\mathbf{D}_S \leftarrow \mathrm{diag}(\{\mathrm{sign}(v_{jj})\}_{j=1}^d);\quad \mathbf{U} \leftarrow \mathbf{U}\mathbf{D}_S;\quad \mathbf{V} \leftarrow \mathbf{V}\mathbf{D}_S;$ — $O(nd + md)$

2. Additional algorithmic details

Stochastic gradient with non-zero covariate effects

In the general case where $\mathbf{B} \neq \mathbf{0}$ and $\mathbf{\Gamma} \neq \mathbf{0}$, in the optimization we must include an explicit update for $\mathbf{B}$ and $\mathbf{\Gamma}$. To this end, we define the first and second elementwise derivatives of the

penalized deviance function with respect to $[\mathbf{\Gamma}, \mathbf{U}]$ and $[\mathbf{B}, \mathbf{V}]$ as

$$\frac{\partial \ell_\lambda}{\partial [\mathbf{\Gamma}, \mathbf{U}]} = \dot{\mathbf{D}}\,[\mathbf{Z}, \mathbf{V}] + \lambda[\mathbf{O}, \mathbf{U}], \qquad \frac{\partial \ell_\lambda}{\partial [\mathbf{B}, \mathbf{V}]} = \dot{\mathbf{D}}^\top [\mathbf{X}, \mathbf{U}] + \lambda[\mathbf{O}, \mathbf{V}],$$

$$\frac{\partial^2 \ell_\lambda}{\partial [\mathbf{\Gamma}, \mathbf{U}]^2} = \ddot{\mathbf{D}}\,[\mathbf{B} * \mathbf{B}, \mathbf{V} * \mathbf{V}] + [\mathbf{O}, \mathbf{\Lambda}], \qquad \frac{\partial^2 \ell_\lambda}{\partial [\mathbf{B}, \mathbf{V}]^2} = \ddot{\mathbf{D}}^\top [\mathbf{X} * \mathbf{X}, \mathbf{U} * \mathbf{U}] + [\mathbf{O}, \mathbf{\Lambda}].$$

At the tth iteration of the proposed adaptive stochastic gradient descent algorithm, the above derivatives can be unbiasedly estimated by generalizing equations (3.15), thus obtaining

$$\hat{\mathbf{G}}^t_{[\Gamma,\mathrm{U}],I:} = (m/m^\star_J)\,\dot{\mathbf{D}}^t_B[\mathbf{Z}_{J,:}, \mathbf{V}^t_{J:}] + \lambda[\mathbf{O}, \mathbf{U}^t_{I:}], \qquad \hat{\mathbf{H}}^t_{[\Gamma,\mathrm{U}],I:} = (m/m^\star_J)\,\ddot{\mathbf{D}}^t_B[\mathbf{Z}_{J:} * \mathbf{Z}_{J:}, \mathbf{V}^t_{J:} * \mathbf{V}^t_{J:}] + [\mathbf{O}, \mathbf{\Lambda}],$$

$$\hat{\mathbf{G}}^t_{[\mathrm{B},\mathrm{V}],J:} = (n/n^\star_I)\,\dot{\mathbf{D}}^{t\top}_B[\mathbf{X}_{I,:}, \mathbf{U}^t_{I:}] + \lambda[\mathbf{O}, \mathbf{V}^t_{J:}], \qquad \hat{\mathbf{H}}^t_{[\mathrm{B},\mathrm{V}],J:} = (n/n^\star_I)\,\ddot{\mathbf{D}}^{t\top}_B[\mathbf{X}_{I:} * \mathbf{X}_{I:}, \mathbf{U}^t_{I:} * \mathbf{U}^t_{I:}] + [\mathbf{O}, \mathbf{\Lambda}].$$

As a result, the joint stochastic update for the regression parameters, $\mathbf{B}$ and $\mathbf{\Gamma}$, and latent variables, $\mathbf{V}$ and $\mathbf{U}$, is obtained as in (3.14):

$$[\mathbf{\Gamma}, \mathbf{U}]^{t+1}_{I:} \leftarrow [\mathbf{\Gamma}, \mathbf{U}]^t_{I:} + \rho_t\,\mathbf{\Delta}^t_{[\Gamma,\mathrm{U}],I:}, \qquad \mathbf{\Delta}^t_{[\Gamma,\mathrm{U}],I:} = -\alpha_t(\bar{\mathbf{G}}^t_{[\Gamma,\mathrm{U}],I:} \,/\, \bar{\mathbf{H}}^t_{[\Gamma,\mathrm{U}],I:}),$$

$$[\mathbf{B}, \mathbf{V}]^{t+1}_{J:} \leftarrow [\mathbf{B}, \mathbf{V}]^t_{J:} + \rho_t\,\mathbf{\Delta}^t_{[\mathrm{B},\mathrm{V}],J:}, \qquad \mathbf{\Delta}^t_{[\mathrm{B},\mathrm{V}],J:} = -\alpha_t(\bar{\mathbf{G}}^t_{[\mathrm{B},\mathrm{V}],J:} \,/\, \bar{\mathbf{H}}^t_{[\mathrm{B},\mathrm{V}],J:}).$$

where $\bar{\mathbf{G}}^t_{[\Gamma,\mathrm{U}],I:}$, $\bar{\mathbf{G}}^t_{[\mathrm{B},\mathrm{V}],J:}$, $\bar{\mathbf{H}}^t_{[\Gamma,\mathrm{U}],I:}$ and $\bar{\mathbf{H}}^t_{[\mathrm{B},\mathrm{V}],J:}$ are the smoothed differentials computed via the exponential averaging in (3.12).

Unknown dispersion parameter

In the case where the dispersion parameter ϕ is unknown and has to be learned from the data, a standard choice in the literature is the Pearson estimator, which is given by

$$\hat{\phi} = \frac{1}{N} \sum_{i=1}^{n} \sum_{j=1}^{m} \frac{(y_{ij} - \hat{\mu}_{ij})^2}{\nu(\hat{\mu}_{ij})/w_{ij}} = \frac{1}{N} \mathbf{1}_n^\top \big[\mathbf{W} * (\mathbf{Y} - \hat{\boldsymbol{\mu}})^2/\nu(\hat{\boldsymbol{\mu}})\big] \mathbf{1}_m.$$

where $N = nm - mp - nq - (n+m)d - 1$ is the effective degrees of freedom of the model, that is the difference between the number of observations and the number of parameters to be estimated. This can be computed *a posteriori* or iteratively refined during the optimization substituting $\hat{\boldsymbol{\mu}}$ with $\boldsymbol{\mu}^t$.

In our optimization routine, we consider a sequential refinement of the dispersion parameter using a smoothed stochastic estimator obtained as

$$\hat{\phi}^{t+1} \leftarrow \frac{1}{N} \frac{nm}{n_I^\star m_J^\star} \mathbf{1}_{n_I^\star}^\top \big[\mathbf{W}_B * (\mathbf{Y}_B - \boldsymbol{\mu}_B^t)^2 / \nu(\boldsymbol{\mu}_B^t)\big] \mathbf{1}_{m_J^\star},$$
$$\bar{\phi}^{t+1} \leftarrow (1-\rho_t)\, \bar{\phi}^t + \rho_t\, \hat{\phi}^{t+1},$$

where $\hat{\phi}^{t+1}$ is the stochastic estimate of ϕ obtained using only the information of the current mini-batch, while $\bar{\phi}^{t+1}$ is a smoothed estimator obtained as the exponential averaging of the current and previous estimates.

Negative Binomial inflation parameter

In the Negative Binomial model, the deviance and variance functions are specified as

$$D_\alpha(y,\mu) = 2w\left[y \log \frac{y}{\mu} - (y+\alpha) \log \frac{y+\alpha}{\mu+\alpha}\right], \quad \nu_\alpha(\mu) = w\mu(1+\mu/\alpha),$$

where α is the shape parameter of the Negative Binomial family. Since the shape parameter $\alpha > 0$ is typically unknown, we need to estimate it from the data. A common choice in the literature is to consider the moment estimator

$$\hat{\alpha} = \frac{\left[\sum_{i=1}^n \sum_{j=1}^m w_{ij} \hat{\mu}_{ij}^2\right]}{\left[\sum_{i=1}^n \sum_{j=1}^m w_{ij} \big\{(y_{ij} - \hat{\mu}_{ij})^2 - \hat{\mu}_{ij}\big\}\right]} = \frac{\mathbf{1}_n^\top (\mathbf{W} * \hat{\boldsymbol{\mu}} * \hat{\boldsymbol{\mu}}) \mathbf{1}_m}{\mathbf{1}_n^\top \big[\mathbf{W} * \{(\mathbf{Y} - \hat{\boldsymbol{\mu}})^2 - \hat{\boldsymbol{\mu}}\}\big] \mathbf{1}_m},$$

where $\hat{\boldsymbol{\mu}}_{ij}$ must be a consistent estimator of the Negative Binomial mean.

Since complete access to the whole data and prediction matrices could be prohibitively expensive in high-dimensional settings, in our optimization scheme, we instead consider the stochastic update

$$\hat{\alpha}^{t+1} \leftarrow \frac{\mathbf{1}_{n_I^\star}^\top (\mathbf{W}_B * \boldsymbol{\mu}_B^t * \boldsymbol{\mu}_B^t) \mathbf{1}_{m_J^\star}}{\mathbf{1}_{n_I^\star}^\top \big[\mathbf{W}_B * \{(\mathbf{Y}_B - \boldsymbol{\mu}_B^t)^2 - \boldsymbol{\mu}_B^t\}\big] \mathbf{1}_{m_J^\star}},$$
$$\bar{\alpha}^{t+1} \leftarrow (1-\rho_t)\, \bar{\alpha}^t + \rho_t \max(\varepsilon, \hat{\alpha}^{t+1}),$$

where $\hat{\alpha}^{t+1}$ is the stochastic estimate of α obtained using only the information of the current mini-batch, while $\bar{\alpha}^{t+1}$ is a smoothed estimator obtained as the exponential averaging of the current and previous estimates, and $\varepsilon > 0$ is a small positive constant introduced to ensure that the final estimate is positive.

3. Simulation setting details

Data generating process

To simulate the data, we use the `R` package `splatter` (Zappia *and others*, 2017), which is freely available on `Bioconductor` (Huber *and others*, 2015). In particular, we use the function `splatSimulateGroups()` to generate the gene-expression matrices.

In our experiments, we considered the following simulation setup: each dataset contains cells from five well-separated types evenly distributed in the sample. The data are also divided into three batches having different expression levels. No lineage or branching effects are considered. The setting-specific dimensions of the gene-expression matrices, n and m, are reported in the paper. Under each simulation setting, we set the simulation parameters specifying the following options in the `splatter` functions `newSplatParams()` and `setParams()`:

- number of genes: `nGenes =` m;
- number of cells: `nCells =` n;
- number of cells per batch: `batchCells = c(`$\lfloor n/3 \rfloor$`,` $\lfloor n/3 \rfloor$`,` $n - 2\lfloor n/3 \rfloor$`);`
- probability of each cell-group: `group.prob = c(0.1, 0.2, 0.2, 0.2, 0.3);`
- probability of gene differential expression in a group: `de.prob = c(0.3, 0.1, 0.2, 0.01, 0.1);`
- probability of gene down-regulation in a group: `de.downProb = c(0.1, 0.4, 0.9, 0.6, 0.5);`
- location of the differential expression factor: `de.facLoc = c(0.6, 0.1, 0.1, 0.01, 0.2);`
- Scale of the differential expression factor: `de.facScale = c(0.1, 0.4, 0.2, 0.5, 0.4).`

Competing methods

We compare the proposed adaptive stochastic gradient descent method for the estimation of generalized matrix factorization models with several state-of-the-art approaches in the literature. In particular, we consider the following models and algorithms.

- ***CMF***: we use the `CMF()` function in the `cmfrec` package (Cortes, 2023), and we specify the following options: `k =` d, `nonneg = TRUE`, `user_bias = FALSE`, `item_bias = FALSE`, `center = FALSE`, `nthreads = 4`, `niter = 1000`.

- ***NMF***: we use the `NMF()` function in the `NMF` package (Gaujoux and Seoighe, 2010), and we specify the following options: `rank =` d, `method = "brunet"`, `seed = "nndsvd"`, `nrun = 1`.

- ***NMF+***: we use the `NNMF()` function in the `NNLM` package (Lin and Boutros, 2020), and we specify the following options: `k =` d, `alpha = 1`, `beta = 1`, `n.threads = 4`, `method = "lee"`, `loss = "mkl"`, `max.iter = 1000`.

- ***AvaGrad***: we use the `glmpca()` function in the *glmPCA* package (Townes *and others*, 2019), and we specify the following options: `L =` d, `fam = "poi"`, `minibatch = "none"`, `optimizer = "avagrad"`, `ctl = list(maxIter = 1000, tol = 1e-05)`.

- ***Fisher***: we use the `glmpca()` function in the *glmPCA* package (Townes *and others*, 2019), and we specify the following options: `L =` d, `fam = "poi"`, `minibatch = "none"`, `optimizer = "fisher"`, `ctl = list(maxIter = 200, tol = 1e-05)`.

- ***NBWaVE***: we use the `newFit()` function in the `NewWave` package (Agostinis *and others*, 2022), and we specify the following options: `K =` d, `commondispersion = TRUE`, `maxiter_optimize = 200`, `stop_epsilon = 1e-05`, `children = 4`.

- ***GFM-AM***: we use the `gfm()` function in the `GFM` package (Liu *and others*, 2023), and we specify the following options: `types = "poisson"`, `q =` d, `offset = FALSE`, `dc_eps = 1e-05`, `maxIter = 200`, `algorithm = "AM"`.

- ***GFM-VEM***: we use the `gfm()` function in the `GFM` package (Liu *and others*, 2023), and we specify the following options: `types = "poisson"`, `q =` d, `offset = FALSE`, `dc_eps = 1e-05`, `maxIter = 200`, `algorithm = "VEM"`.

- ***COAP***: we use the `COAP_RR()` function in the `COAP` package (Liu and Zhong, 2024), and we specify the following options: `Z = X`, `q =` d, `epsELBO = 1e-05`, `maxIter = 100`, `joint_opt_beta = FALSE`, `fast_svd = FALSE`.

- ***AIRWLS***: we use the `cpp.fit.airwls()` function in the `sgdGMF` package, which implement the AIRWLS algorithm of Kidziński *and others* (2022) and Wang and Carvalho (2023), and we specify the following options: `ncomp =` d, `familyname = "poisson"`, `linkname = "log"`, `lambda = c(0,0,1,0)`, `maxiter = 200`, `nsteps = 1`, `stepsize = 0.2`, `eps = 1e-08`, `nafill = 1`, `tol = 1e-05`, `damping = 0.001`, `parallel = TRUE`, `nthreads = 4`.

- ***Newton***: we use the `cpp.fit.newton()` function in the `sgdGMF` package, which implements the quasi-Newton algorithm of Kidziński *and others* (2022), and we specify the following options: `ncomp =` d, `familyname = "poisson"`, `linkname = "log"`, `lambda = c(0,0,1,0)`, `maxiter = 200`, `stepsize = 0.2`, `eps = 1e-08`, `nafill = 1`, `tol = 1e-05`, `damping = 0.001`, `parallel = TRUE`, `nthreads = 4`.

- ***aSGD***: we use the `cpp.fit.bsgd()` function in the `sgdGMF` package, and we specify the following options: `ncomp =` d, `familyname = "poisson"`, `linkname = "log"`, `lambda = c(0,0,1,0)`, `maxiter = 500`, `rate0 = 0.01`, `size1 = 100`, `size2 = 20`, `eps = 1e-08`, `nafill = 1`, `tol = 1e-05`, `damping = 1e-03`.

The results of *AIRWLS*, *Newton*, and *aSGD* with the negative binomial likelihood were obtained substituting `familyname = "poisson"` with `familyname = "NegativeBinomial"`. It is worth mentioning that the original `R` implementation of the *AIRWLS* and *Newton* methods can be found in the `gmf` package (Kidziński *and others*, 2022). However, such an implementation does not permit the inclusion of gene-specific intercepts and covariate effects, say $\boldsymbol{\gamma}_i^\top \boldsymbol{z}_j$ in our notation, and also it does not allow for parallel computing in Windows operating systems. Therefore, we performed the benchmarking experiments using the `R/C++` implementation in the proposed `sgdGMF` package.

All the options not specified here are left to default values. For all the `R` scripts we used for running the simulation and plotting the results, please refer to the GitHub repository `https://github.com/alexandresegers/sgdGMF_Paper`.

4. Supplementary Figures and Tables

Supplementary Table 1 summarizes commonly used dispersion exponential family distributions, together with their key characteristics, including the support, canonical link, variance function, and deviance function. The distributions listed constitute a non-exhaustive set of likelihood models that can be accommodated within the GMF framework considered in this work and are all implemented in the accompanying `R` package `sgdGMF`.

Supplementary Table 1. Exponential family laws along with their support space, canonical link (g_c), variance (ν), and rescaled deviance ($D/2a$) functions. Here, we denote by $\alpha > 0$ the variance parameter of the Negative Binomial distribution.

Distribution	Support	$g_c(\mu)$	$\nu(\mu)$	$D(y,\mu)/2a(\phi)$
Gaussian	$\mathbb{R}$	μ	1	$(y-\mu)^2/2$
Gamma	$\mathbb{R}_+$	$1/\mu$	μ^2	$(y-\mu)/\mu - \log(y/\mu)$
Inv. Gauss.	$\mathbb{R}_+$	$1/\mu^2$	μ^3	$(y-\mu)^2/(2y\mu^2)$
Poisson	$\mathbb{N}_+$	$\log(\mu)$	μ	$y\log(y/\mu) - (y-\mu)$
Bernoulli	$\{0,1\}$	$\log\{\mu/(1-\mu)\}$	$\mu(1-\mu)$	$y\log(y/\mu) + (1-y)\log\{(1-y)/(1-\mu)\}$
Neg. Binom.	$\mathbb{N}_+$	$\log(\mu)$	$\mu(1+\mu/\alpha)$	$y\log(y/\mu) - (y+\alpha)\log\{(y+\alpha)/(\mu+\alpha)\}$

Simulation studies: additional results

In Section 5, we compared the algorithms implemented in the `R` package `sgdGMF` under a Poisson likelihood with several state-of-the-art methods from the literature. Here, we present an additional simulation study aimed at comparing the computational efficiency and goodness-of-fit of the `sgdGMF` implementation under Poisson and Negative Binomial GMF models, in order to assess potential advantages of one likelihood specification over the other.

We adopt the same simulation settings described in Section 5, focusing on the *AIRWLS*, *Newton*, and *aSGD* algorithms as implemented in `sgdGMF` under both Poisson and Negative Binomial likelihoods.

Supplementary Fig. 1 reports the execution time (in seconds) and peak memory usage (in megabytes) across the two simulation settings and the six methods considered. Supplementary Fig. 2 summarizes the corresponding out-of-sample error, out-of-sample deviance, average silhouette, and neighborhood purity.

Overall, *aSGD* shows comparable computational performance under both likelihoods in terms of execution time and memory consumption. In contrast, *AIRWLS* and *Newton* exhibit some sensitivity to the likelihood choice: in setting A, the Negative Binomial model converges faster than the Poisson model, leading to reduced computation time, whereas no substantial differences are observed in setting B.

In terms of signal reconstruction, Poisson models consistently achieve lower logarithmic root mean squared error, while Negative Binomial models yield lower deviance. Moreover, Negative Binomial models attain higher silhouette and neighborhood purity scores, indicating improved separation of cell lines in the latent space compared to Poisson models within the `sgdGMF` framework.

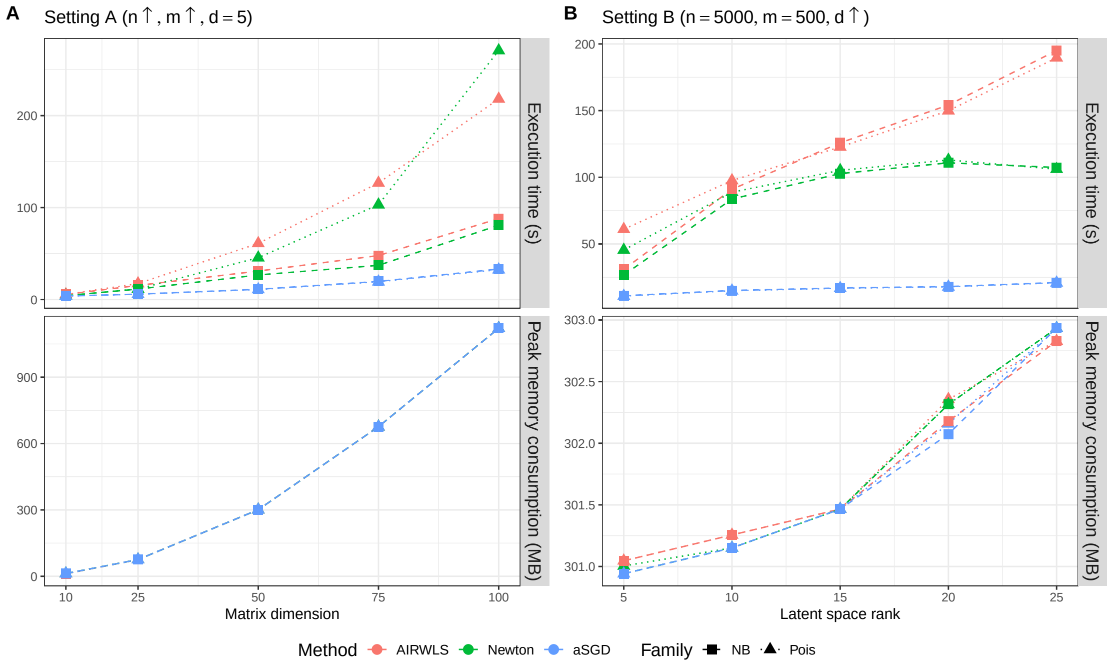

Supplementary Figure 1. Summary statistics of the simulation experiments described in Section 5.1. The columns correspond to simulation settings A (left) and B (right). The rows correspond to the elapsed execution time in seconds (top) and the peak memory consumption in megabytes (bottom).

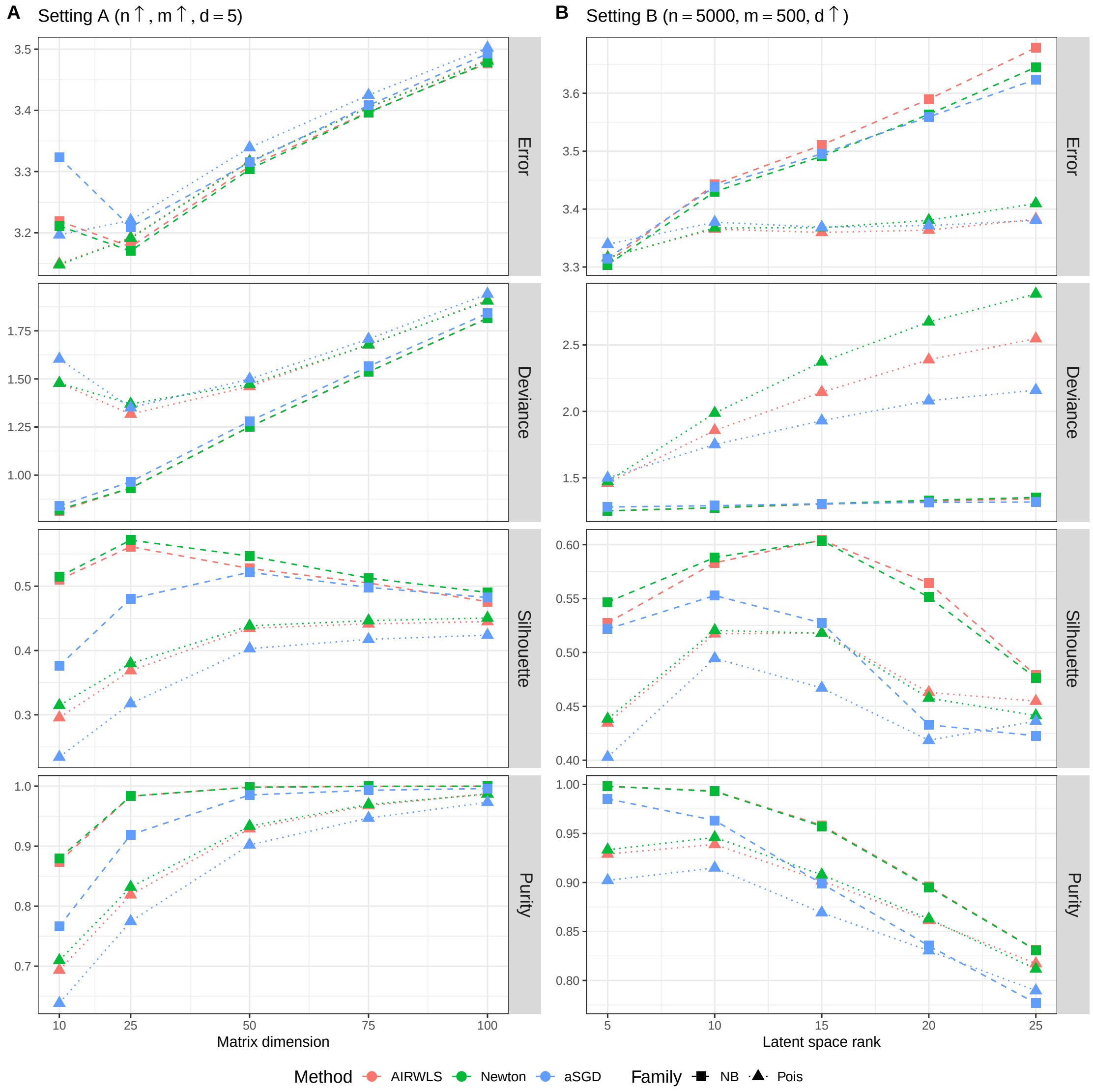

Supplementary Figure 2. Summary statistics of the simulation experiments described in Section 5.1. The columns correspond to simulation settings A (left) and B (right). The rows correspond to four goodness-of-fit measures. From top to bottom: the out-of-sample relative logarithmic root mean squared error, the out-of-sample relative residual deviance, the silhouette evaluated on a 2-dimensional tSNE projection of the latent space, and the neighborhood purity of the true cell-type evaluated on the original latent space.

Arigoni dataset: additional results

In addition to the results presented in Section 6.1, we also conducted further numerical explorations to assess the sensitivity of the results obtained under the proposed *aSGD* algorithm in the Arigoni dataset. In particular, we are interested in inspecting the quality of the estimated latent space representation for varying numbers of latent factors and high variable genes.

Supplementary Fig. 3 shows the 2-dimensional tSNE embeddings of the estimated latent factors for all the combinations of latent space ranks $\{5, 10, 30\}$ and high-variable genes $\{100, 200, 500, 1000, 1500, 2000\}$. Overall, the results are quite robust in the different scenarios. Specifically, we observe that even with a small rank and a low number of high-variable genes, the *aSGD* algorithm is able to recover the most important features of the dataset, and in particular, it successfully recovers most of the cell types. Increasing the latent space rank and the number of high-variable genes has the effect of improving the separation between cell types A549 and CCL-185-IG, without helping much in the separation for all the other cell types.

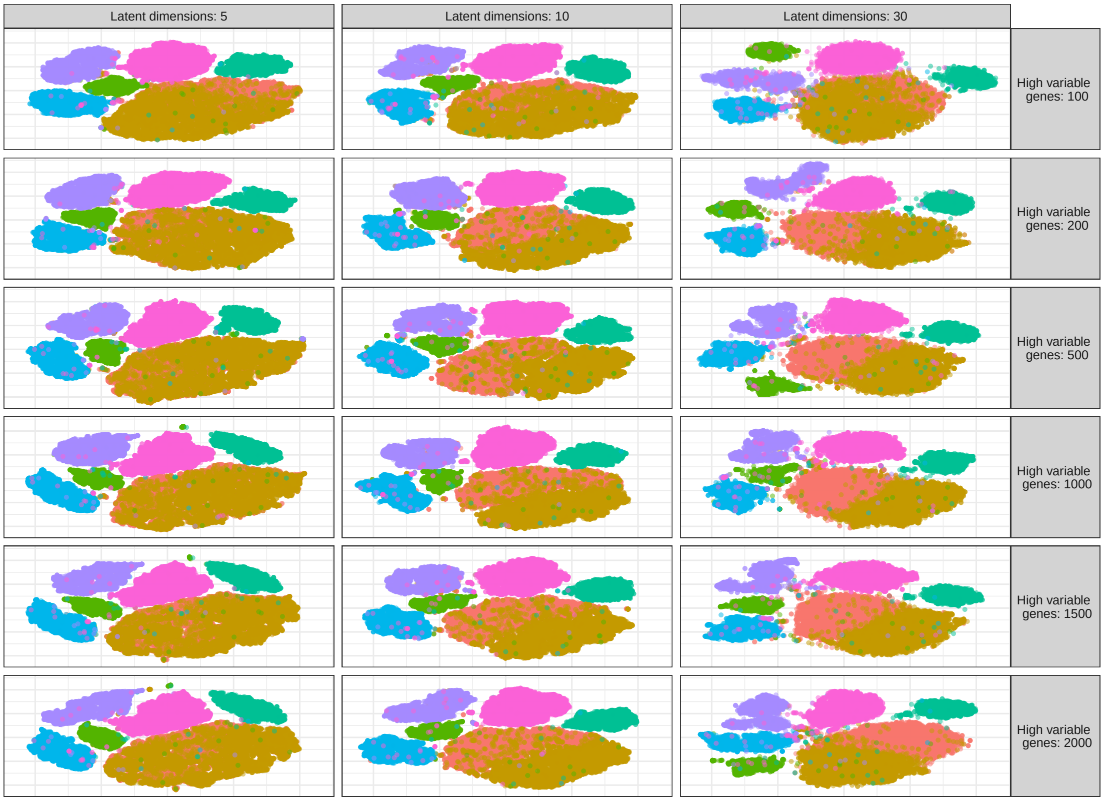

Supplementary Figure 3. Comparison of 2-dimensional tSNE embeddings obtained from the latent factors estimated via *aSGD* with a varying number of highest variable genes and a varying number of latent factors.

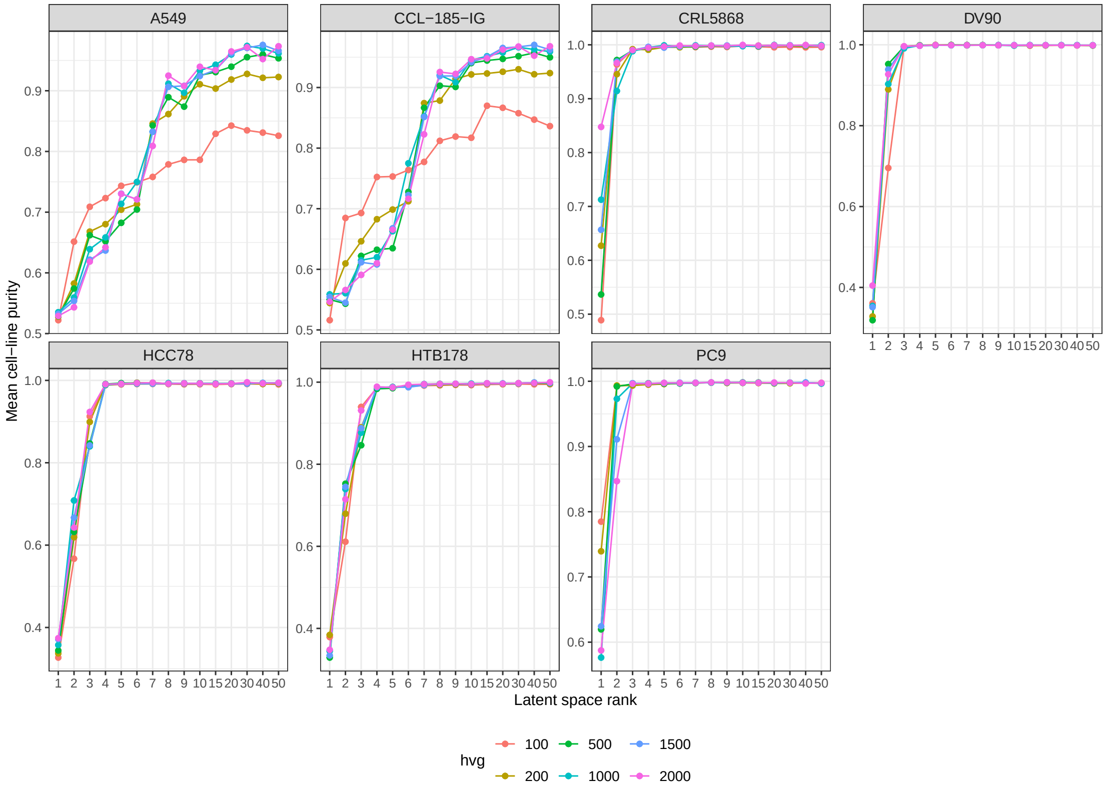

Supplementary Figure 4. Comparison of average neighbor purity stratified by cell type obtained from the latent factors estimated via *aSGD* with a varying number of highest variable genes.

Supplementary Fig. 4 shows the mean neighborhood purity stratified by cell type for an increasing number of latent space ranks (ranging from 1 to 50) and high-variable genes (ranging from 100 to 2000). This representation clearly highlights how there is no significant gain from adding more than 500 high-variable genes in the analysis. Moreover, we can also observe that after roughly latent space rank 10, the gain of adding additional latent factors is negligible and contributes only to the separation of cell types A549 and CCL-185-IG, which actually are two cell types very difficult to separate in this framework, as discussed also in Arigoni *and others* (2024).

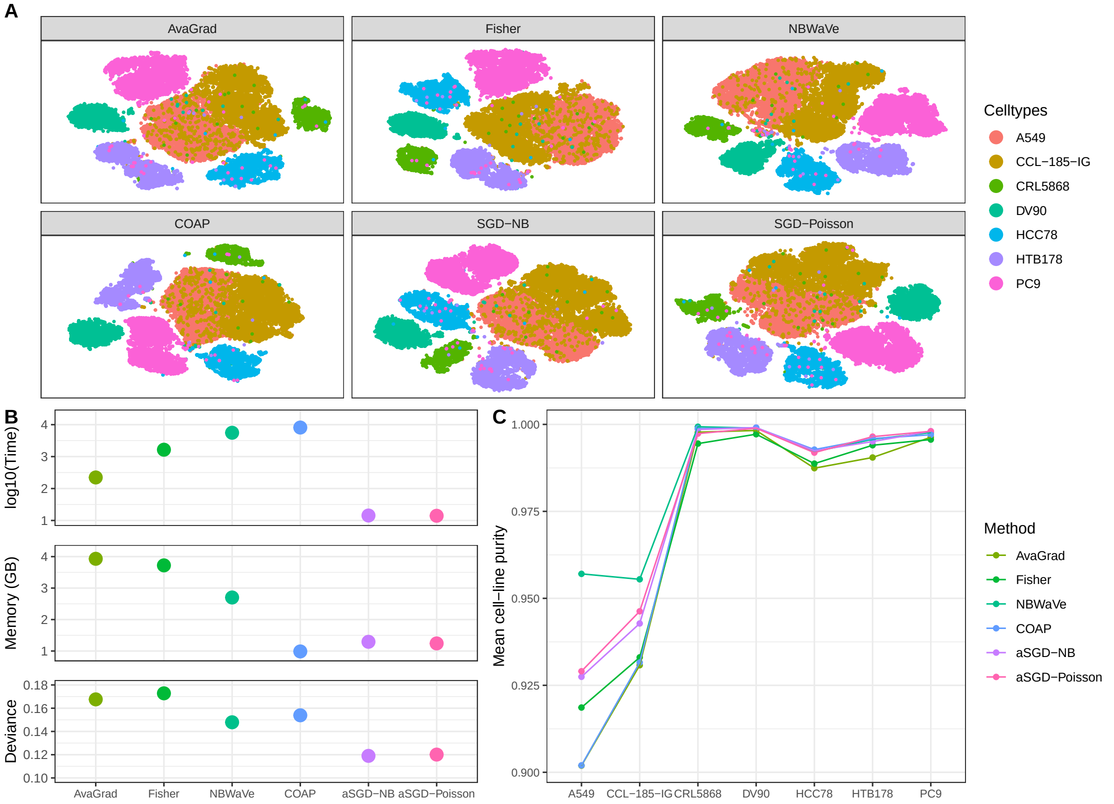

Supplementary Figure 5. Comparison of *aSGD* (under both Poisson and Negative Binomial likelihoods) with *NBWaVE*, *glmPCA*, and *COAP* on the Arigoni dataset. All methods are estimated with a latent space rank of dimension 15, which was suggested by model selection criteria. A) 2-dimensional tSNE embeddings of the estimated latent factors. B) From top to bottom: $\log_{10}$ execution time in seconds, peak memory consumption in gigabytes, out-of-sample deviance. C) Average neighbor purity stratified by cell type.

As a final numerical assessment, we compare the proposed *aSGD* algorithm, under both Poisson and Negative Binomial likelihoods, with *NBWaVE* (Agostinis *and others*, 2022), *glmPCA* (Townes *and others*, 2019), and *COAP* (Liu and Zhong, 2024) on the Arigoni dataset (Arigoni *and others*, 2024), using a common latent space rank equal to 15, which is the value suggested by model selection criteria discussed in Section 6.1. The results of such a comparison are reported in Supplementary Fig. 5. The 2-dimensional tSNE embeddings (panel A) show no significant differences between all methods in terms of cell type separation. Such a result is also

confirmed by the analysis of the average stratified neighbor purity (panel C), which presents a similar magnitude across all models and for all cell types. Such an equivalence in terms of cell type separation does not reflect directly on the out-of-sample deviance (panel B); in fact, *aSGD* systematically reaches lower out-of-sample deviance when predicting missing values. This is probably due to *NBWaVE*, *glmPCA*, and *COAP* requiring imputation of missing values prior to computation of the latent structure, while *aSGD* can deal with missing values internally. From a computational viewpoint (panel B), *aSGD* is orders of magnitude faster compared to the other methods, while being also very parsimonious in terms of peak memory usage, reaching the same level of memory consumption as *COAP*. The results obtained with *aSGD* appear very consistent across all metrics and likelihood specifications, not showing outstanding differences between Poisson and Negative Binomial families on this dataset. Therefore, we can conclude that *aSGD* is a fast alternative to *NBWaVE*, *glmPCA*, and *COAP*, while having similar performance in terms of signal extraction and cell type separation in the latent space.

TENxBrainData: additional results

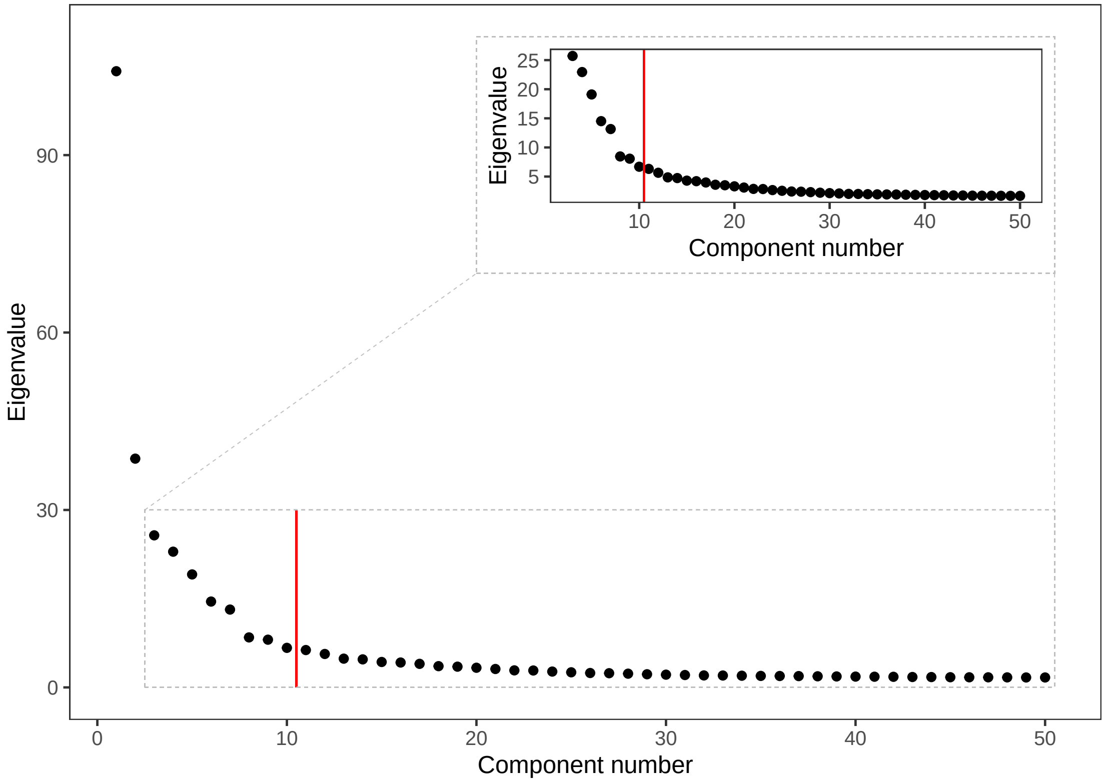

Supplementary Figure 6. Eigenvalues of the latent directions ordered in descending values for the TENxBrainData (Lun and Morgan, 2023). This plot, also called a scree-plot can be used for rank selection by looking for an elbow in the curve. Here, 10 latent factors were chosen to be used in the case study.

References

Agostinis, Federico, Romualdi, Chiara, Sales, Gabriele and Risso, Davide. (2022). NewWave: a scalable R/Bioconductor package for the dimensionality reduction and batch effect removal of single-cell RNA-seq data. *Bioinformatics* **38**(9), 2648–2650.

Arigoni, Maddalena, Ratto, Maria L, Riccardo, Federica, Balmas, Elisa, Calogero, Lorenzo, Cordero, Francesca, Beccuti, Marco, Calogero, Raffaele A and Alessandri, Luca. (2024). A single cell RNAseq benchmark experiment

embedding "controlled" cancer heterogeneity. *Scientific data* **11**, 159.

Cortes, David. (2023). *cmfrec: Collective Matrix Factorization for Recommender systems*. R package version 3.5.1-1.

Gaujoux, Renaud and Seoighe, Cathal. (2010). A flexible r package for nonnegative matrix factorization. *BMC Bioinformatics* **11**(1), 367.

Huber, W., Carey, V. J., Gentleman, R., Anders, S., Carlson, M., Carvalho, B. S., Bravo, H. C., Davis, S., Gatto, L., Girke, T., Gottardo, R., Hahne, F., Hansen, K. D., Irizarry, R. A., Lawrence, M., Love, M. I., MacDonald, J., Obenchain, V., Ole's, A. K., Pag'es, H., Reyes, A., Shannon, P., Smyth, G. K., Tenenbaum, D., Waldron, L. *and others*. (2015). Orchestrating high-throughput genomic analysis with Bioconductor. *Nature Methods* **12**(2), 115–121.

Kidziński, Ł ukasz, Hui, Francis K. C., Warton, David I. and Hastie, Trevor J. (2022). Generalized matrix factorization: efficient algorithms for fitting generalized linear latent variable models to large data arrays. *Journal of Machine Learning Research* **23**(291), 1–29.

Lin, Xihui and Boutros, Paul C. (2020). *NNLM: Fast and Versatile Non-Negative Matrix Factorization*. R package version 0.4.4.

Liu, Wei, Lin, Huazhen, Zheng, Shurong and Liu, Jin. (2023). Generalized factor model for ultra-high dimensional correlated variables with mixed types. *Journal of the American Statistical Association* **118**(542), 1385–1401.

Liu, Wei and Zhong, Qingzhi. (2024). High-dimensional covariate-augmented overdispersed Poisson factor model. *Biometrics* **80**(2), Paper No. ujae031, 12.

Lun, Aaron and Morgan, Martin. (2023). TENxBrainData: Data from the 10x1.3

million brain cell study. doi:10.18129/B9.bioc.TENxBrainData, R package version 1.22.0, https://bioconductor.org/packages/TENxBrainData.

Risso, Davide, Perraudeau, Fanny, Gribkova, Svetlana, Dudoit, Sandrine and Vert, Jean-Philippe. (2018). A general and flexible method for signal extraction from single-cell rna-seq data. *Nature communications* **9**(1), 284.

Townes, F. William, Hicks, Stephanie C., Aryee, Martin J. and Irizarry, Rafael A. (2019). Feature selection and dimension reduction for single-cell RNA-Seq based on a multinomial model. *Genome Biology* **20**, 1–16.

Wang, Liang and Carvalho, Luis. (2023). Deviance matrix factorization. *Electronic Journal of Statistics* **17**(2), 3762–3810.

Zappia, Luke, Phipson, Belinda and Oshlack, Alicia. (2017). Splatter: simulation of single-cell rna sequencing data. *Genome Biology* **18**(1), 174.

[*Received August 1, 2010; revised October 1, 2010; accepted for publication November 1, 2010*]
